# NOV-002, A Glutathione Disulfide Mimetic, Suppresses Tumor Cell Invasion and Metastasis

**DOI:** 10.4172/2157-2518.S7-002

**Published:** 2013-04-30

**Authors:** Kiranmai Gumireddy, Anping Li, Lili Cao, Jinchun Yan, Lin Liu, Xiaowei Xu, Christopher Pazoles, Qihong Huang

**Affiliations:** 1 The Wistar Institute, 3601 Spruce Street, Philadelphia, PA 19104, USA; 2 Central labortary, Shandong Provincial Qianfoshan Hospital, Jinan, Shandong 250014, P. R. China; 3 University of Washington Medical Center, 1959 N.E. Pacific Street, Seattle, WA 98195, USA; 4 Department of Oncology, Shandong Cancer Hospital and Institute, Jinan, Shandong 250117, P. R. China; 5 Department of Pathology and Laboratory Medicine, Hospital of the University of Pennsylvania, Philadelphia, PA 19104, USA; 6 Novelos Therapeutics Inc. One Gateway Center, Newton, MA 02458, USA

## Abstract

Metastasis is the major cause of death in cancer. Most therapies currently in the clinic aim to eradicate primary tumor, but do not have ideal effects on metastasis. The lack of effective therapy in metastasis prevention and treatment results in high mortality rate in cancer patients with advanced diseases. Here we report the oxidized glutathione small molecule compound NOV-002 reduces cancer cell invasion *in vitro* and metastasis in an animal model in combination with chemotherapy drug gemcitabine. NOV-002 regulates cell signaling pathways by suppressing ErbB2 and PI3K phosphorylation and subsequent inhibition of Akt and RhoA activation. Our results suggest that NOV-002 affects cell signaling pathways that are critical for invasion and metastasis and can potentially be effective in metastasis treatment in combination of other chemotherapies.

## Introduction

Metastasis is the formation of tumors at distant sites following the spread of cancer from a primary site [1–3]. When cancer is detected before it has spread, it can often be treated successfully with surgery, radiation and chemotherapy. However when it is detected after it has metastasized, treatments are much less successful [2]. Furthermore, due to the lack of diagnostic tools, many patients in whom there is no evidence of metastasis at the time of their initial diagnosis, develop metastases later [4]. Metastasis, rather than the primary tumor, is thus responsible for the majority of cancer deaths, and has consequently become the most feared aspect of cancer [5].

Metastasis occurs *via* a multi-step process requiring the coordinated action of a number of genes [6–8]. The completion of this complex journey requires coordination among the genes responsible for each step of the metastatic process. It has been shown that multiple cell signaling pathways either promote or suppress metastasis [6–8]. ErbB2 signaling network is one the pathway responsible for metastasis. ErbB2 is a member of subclass I of the receptor tyrosine kinase superfamily [9–12]. ErbB2 plays important roles in human cancer. The expression or activation of ERBB2 is altered in many epithelial tumors such as breast, ovarian, gastric and non-small-cell lung cancers [13–16]. ErbB2 is also critical in tumor metastasis [17–21]. ErbB2 activation leads to the stimulation of many cell signaling pathways such as phophatidylinositol 3-kinase (PI3K)-AKT pathways, STAT pathways, Mitogen-Activated Protein Kinase (MAPK) pathways and SRC tyrosine kinase pathways [9–12]. The signaling pathway of ErbB2 and its downstream signaling molecules have been heavily investigated; however, the regulation of ErbB2 by redox homeostasis and its impacts on cellular processes have not been extensively studied.

NOV-002, composed of the disodium salt of glutathione disulfide in a 1,000:1 ratio with cisplatin, is a mimetic of oxidized glutathione (GSSG) that regulates oxidative signaling. It has been shown that NOV-002 modulates cellular redox balance in human HL-60 cells [22,23]. It reduces cell surface protein thiols which has been shown to serve as sensors for redox conditions and regulate cell signaling pathways in a variety of cellular processes [22,23]. NOV-002 exerts pleiotropic effects on cell signaling pathways. It activates the JNK pathway which is responsible for cell proliferation [22,23]. It has also been shown that GSSG can activate MAPK signaling pathway that leads to cell death [24,25]. The effects of GSSG in cell signaling regulation are cell type specific.

Although redox regulation by NOV-002 results in pleiotropic effects on cell functions, its impacts on metastasis have not been studied. Here we showed that NOV-002 regulates ErbB2-PI3K signaling pathway and suppresses cell invasion *in vitro* and metastasis *in vivo* in combination with chemotherapy drug gemcitabine.

## Materials and Methods

### Antibodies and reagents

Anti-human Akt, phospho-Akt-(Thr-308), were purchased from Cell Signaling Technology (Beverly, MA); ErbB2, phosphorylated Tyrosine, clone 4G10, PI3 kinase p85, and RhoA activation assay kit were purchased from Upstate Biotechnology (Lake Placid, NY).

### Cell culture

Human cancer cell lines HCT15, MDA-MB-436, A549, SKOV3 and mouse breast cancer cell line 4T1 were cultured in DMEM, HCT116 cells in McCoy 5A medium, Colo205 in RPMI medium, with 10% fetal bovine serum (FBS). All cells were grown at 37°C with 5% CO2.

Rho activity was measured by affinity precipitation of GTP-Rho with Rhotekin-agarose beads (Rho activation assay kit, Upstate Biotechnology, Lake Placid, NY, USA) according to themanufacturer’s instructions.

### Immunoblotting and immunoprecipitation

Immunoblotting and immunoprecipitation were performed as described (26, 27). Briefly, cells were lysed in RIPA buffer. Equal amounts of protein for each sample were electrophoresed through a 10% SDS-PAGE gel and blotted onto a hybond nitrocellulose membrane from Amersham (Piscataway, NJ). The membrane was blocked with 5% nonfat milk solution and probed with appropriate antibodies. Proteins were detected with enhanced chemiluminiscence (ECL, Amersham Pharmacia Biotech).

Cell lysates were incubated with antibodies for immunoprecipitation. The immunocomplexes were separated on polyacrylamide gels by SDS-PAGE. The Western blotting was performed as described above.

The Western blots were scanned and densities of bands were measured by Image-Pro Plus version 6.0 software. The relative density was calculated by dividing the percent value for each sample by the percent value of respective controls. Data represent mean ± SD of three independent experiments.

### Invasion assay

Matrigel invasion assays were performed as described [26] using transwell chambers (8 μM pore size; Costar). Briefly, transwells were coated with growth factor reduced Matrigel. Subconfluent cell cultures were serum starved for 24 h and cell suspension was added to the upper chamber. NOV-002 (10 μM, 30 μM, 100 μM, 300 μM, 1 mM) was added to the cell suspension. Complete medium was added to the bottom wells of the chambers. Cells migrated to the lower surface of the filters were fixed and stained. Images of three different X10 fields were captured from each membrane and the number of invading cells was counted. The mean of triplicate assays for each experimental condition was used.

### Orthotopic xenograft mouse model

To establish the animal model for metastasis and NOV-002 treatment, 1×10^6^ 4T1-Luc cells were orthotopically transplanted into the mammary fat pads of 7-week-old Balb/c mice. The treatment started on the second day of the cell transplantation. Mice were divided into four groups with 10 mice per group. In the control group, PBS (50 μl per mouse) were injected via intraperitoneal (IP) everyday for the first two weeks, and then five days a week for three weeks. In the NOV-002 treatment group, NOV-002 (100 mg/day/kg) were injected via IP for the first two weeks, and then five days a week for three weeks. In the gemcitabine treatment group, gemcitabine (5 mg/day/kg) were injected via IP once a week for a total of five weeks. In the group of NOV-002 and gemcitabine combination treatment group, NOV-002 (100 mg/day/kg) were injected via IP for the first two weeks, and then five days a week for three weeks; gemcitabine (5 mg/day/kg) were injected via IP once a week for a total of five weeks. Mice bearing luciferase positive tumors were imaged post-transplantation using the bioluminescence Xenogen IVIS system as described [28].

### ErbB2 expression knockdown by short hairpin RNAs

To generate retroviruses, human embryonic kidney (HEK) 293T cells were cotransfected with 1.0 μg of a plasmid expressing Retro ErbB2 shRNA or nontarget shRNA and 0.5 μg of SVA packaging plasmid using Lipifectamine 2000(Invitrogen) according to the manufacturer’s specifications. Viruses were harvested after 48 h by filtering through 0.45 μm filters (Millipore). Colo205 and A549 cells were transduced with the generated retroviruses and the knockdown efficiency was determined by immunobloting.

### Cell viability assay

Cell viability was quantified using MTT Cell Growth Assay kit (Millipore) according to the manufactures protocol. Briefly, exponentially growing cells were seeded in triplicate in 96-well flat-bottomed tissue culture plates at 5×10^3^ cells/well in 0.1 ml medium and treated with 1 mM final concentration of NOV-002. Control wells contained medium without drug. After 72 h treatment cells were incubated with 0.5 mg/mL MTT (3-(4,5-dimethylthiazol-2-yl)-2,5-diphenyl tetrazolium bromide) for 4 h at 37°C. At the end of the incubation, the purple MTT formazan crystals were dissolved by adding 0.1 mL isopropanol with 0.04 N HCl. Cell viability was then measured at 570 nm using a multi-well plate reader (Wallac Victor3, PerkinElmer). The ratio of cell viability was calculated with the equation as follows: (average absorbance of the 1 mM compound)/(average absorbance of the control) ×100. Data represent mean ± SD of three independent experiments.

### Statistical analysis

Comparisons between the means of two data points were performed using the student t test. A p<0.05 was considered statistically significant.

## Results

**Figure 1:**
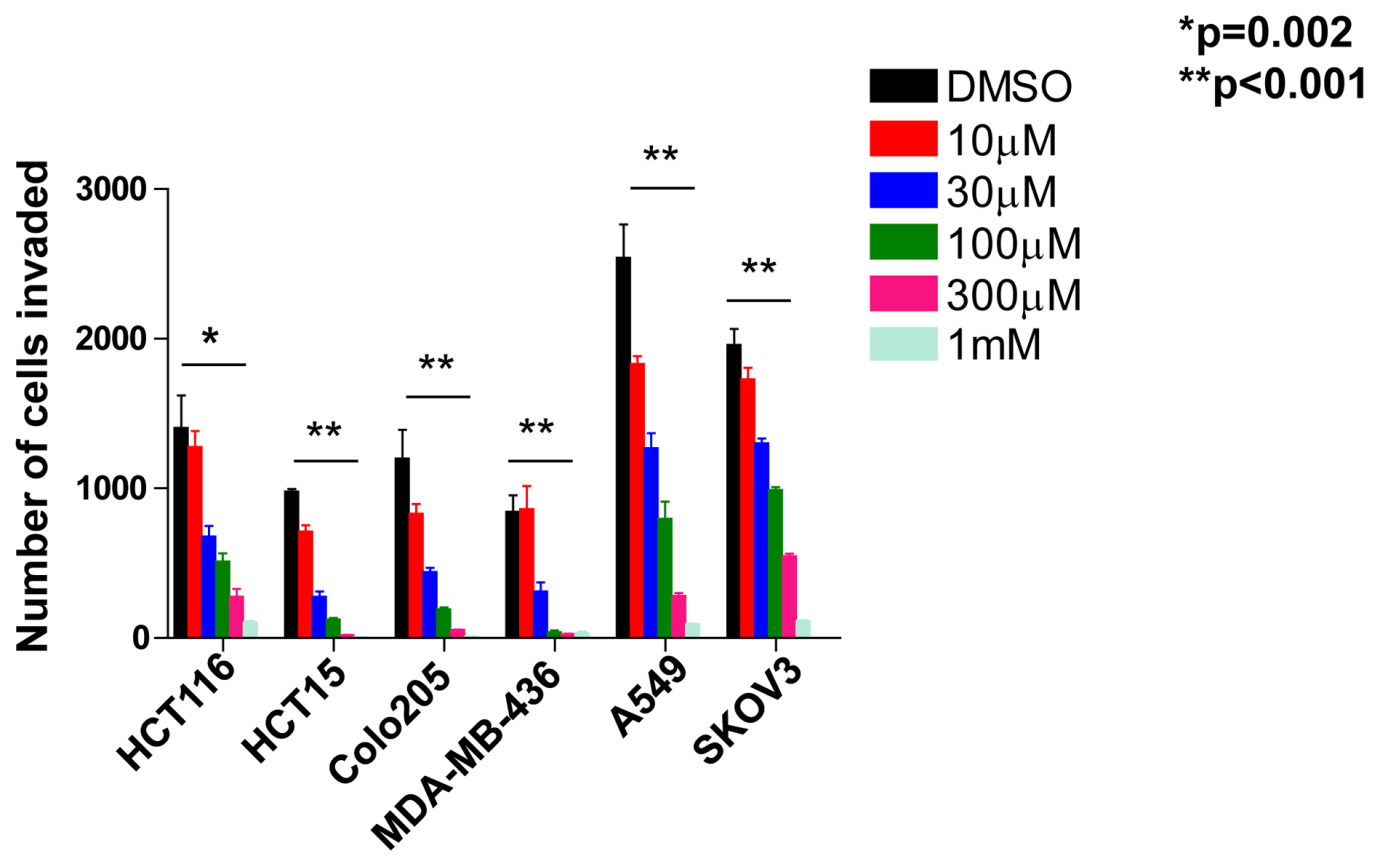
NOV-002 suppresses cell invasion. The graph represents the number of invaded cells from human tumor cell lines after the treatment of various doses of NOV-002 or control (water) in invasion assay.

We determined the effects of NOV-002 on cell invasion in cancer cell lines of various types. We found that NOV-002 suppresses cell invasion over in colon cancer cell HCT15, HCT116 and Colo205, breast cancer cell MDA-MB-436, lung cancer cell A549 and ovarian cancer cell SKOV3, in a dose-dependent manner (Figure 1 and Supplementary Figure 1). The percentage of suppression is over 90% with 1 mM NOV-002 treatment in all these cell lines (Figure 1). The effect on invasion by NOV-002 is not due to cytotoxicity as 1 mM of NOV-002 does not affect cell death in these six cancer cell lines (Supplementary Figure 2). These results suggest that NOV-002 may affect molecules that are critical to invasion process.

**Figure 2:**
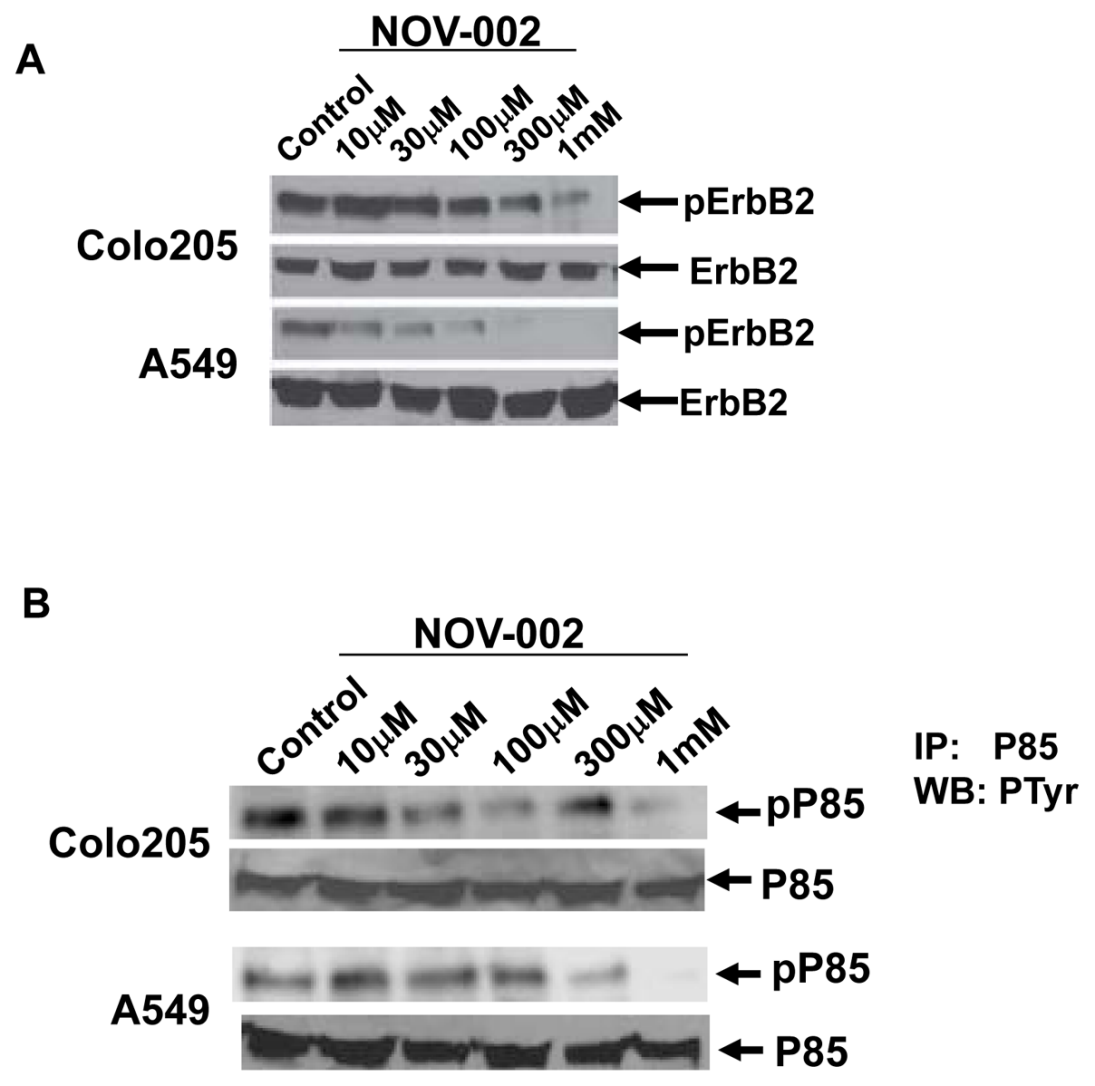
NOV-002 suppresses the phosphorylation of ErbB2 and PI3K subunit P85. (A) Immunoblots of phosphorylated ErbB2 and total ErbB2 in Colo205 and A549 cells with the treatment of various doses of NOV-002 or control (water). (B) Immunoblots of phosphorylated PI3K subunit P85 and total P85 in Colo205 and A549 cells with the treatment of various doses of NOV-002 or control (water). P85 phosphorylation was determined by immunoprecipitation using a P85 antibody, followed by immunoblot analysis with an antibody specific for phosphotyrosine.

It is suggested that the effects of NOV-002 on cell functions may be through its effects on cell surface proteins since oxidized glutathione (GSSG) does not passively cross cell membranes [29]. We determined the effects of NOV-002 on ErbB2 activation because ErbB2 has been shown to regulate cell invasion in cancer [17–21] and is regulated by redox mechanisms [26]. The level of ErbB2 phosphorylation was significantly reduced by 1 mM NOV-002 treatment in A549 and Colo205 cells whereas total ErbB2 expression level was not affected, indicating NOV-002 suppresses ErbB2 activation (Figure 2A and Supplementary Figure 3). ErbB2 regulates multiple cell signaling pathways including PI3K pathway, MAPK pathway and SRC pathway [10,12]. We examined the effects of NOV-002 on PI3K activity which has been shown to play an important role in cell invasion and metastasis [30–32]. It has been shown that the phosphorylation status of P85 subunit of PI3K correlates with PI3K activity [33,34]. P85 phosphorylation was determined by immunoprecipitation using a P85 antibody, followed by immunoblot analysis with an antibody specific for phosphotyrosine. The level of P85 phosphorylation is dramatically reduced by the treatment of 1 mM of NOV-002, indicating that NOV-002 suppresses PI3K activation (Figure 2B and Supplementary Figure 3). In order to determine whether ErbB2 is critical for cell invasion, we knocked down ErbB2 in A549 and Colo205 cells using short hairpin RNAs (shRNAs). Western blot analysis confirmed that ErbB2 expression is significantly reduced following the introduction of shRNAs (Supplementary Figure 4). Knockdown of ErbB2 resulted in the decrease of invasion in A549 and Colo205 cells (Supplementary Figure 5). These results indicated that knockdown of ErbB2 recapitulated the invasion suppression phenotype induced by NOV-002 treatment.

**Figure 3:**
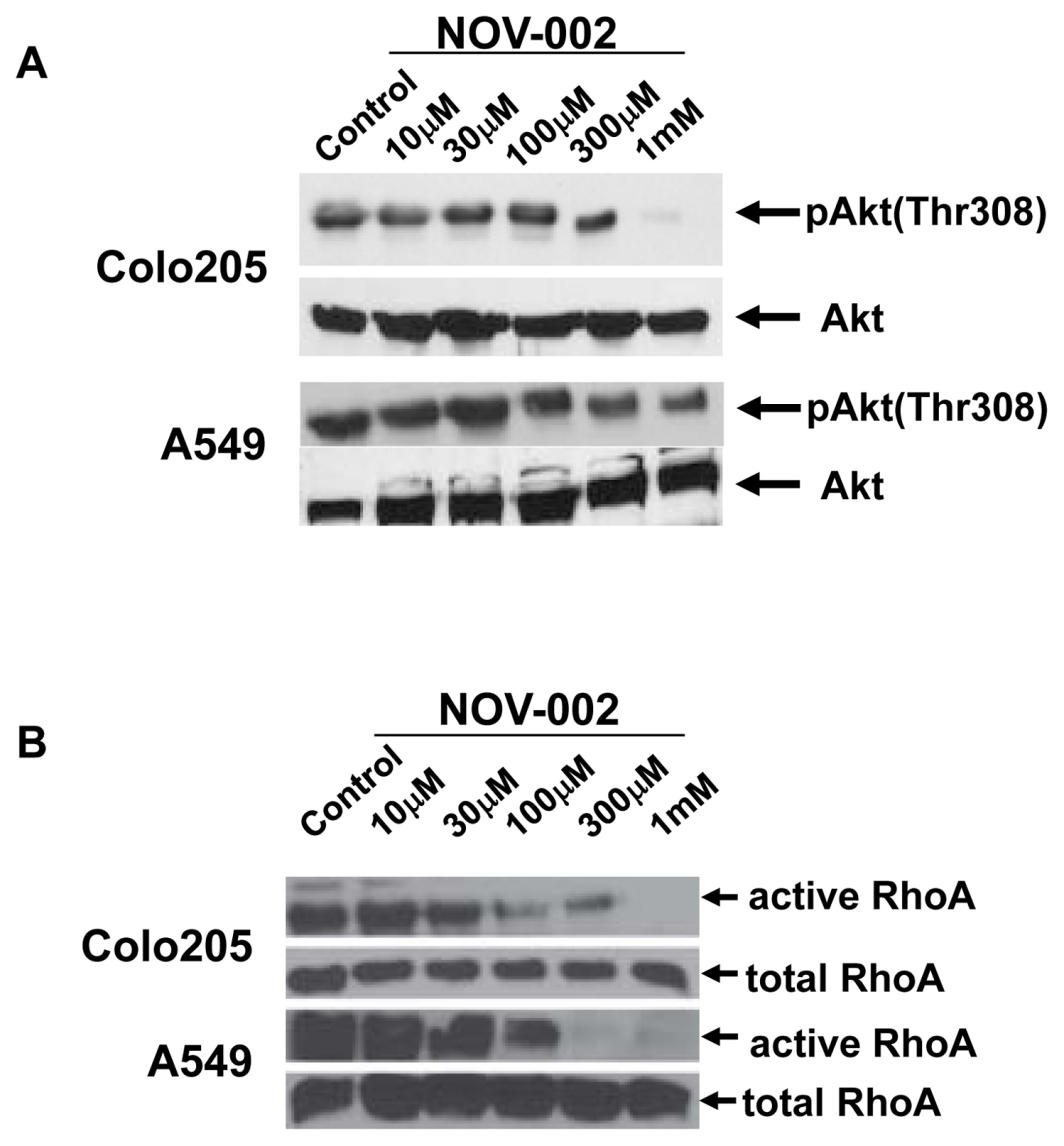
NOV-002 suppresses the activation of Akt and RhoA. (A) Immunoblots of phosphorylated Akt and total Akt in Colo205 and A549 cells with the treatment of various doses of NOV-002 or control (water). (B) Immunoblots of active RhoA and total RhoA in Colo205 and A549 cells with the treatment of various doses of NOV-002 or control (water).

Serine-threonine kinase Akt and RhoA are two major signaling molecules downstream of PI3K activation that play critical role in cell invasion [35]. Akt is activated by phosphorylation at Thr308 and/or Ser473 site [36,37]. The active form of Akt is reduced by NOV-002 treatment (Figure 3A and Supplementary Figure 6) whereas total Akt expression levels are similar with or without NOV-002 treatment. Affinity precipitation of active RhoA from the cell extracts of A549 and Colo205 revealed significant lower active RhoA expression after NOV-002 treatment whereas total RhoA expression does not change with or without NOV-002 treatment (Figure 3B and Supplementary Figure 6). These results suggest that the activation of PI3K downstream signaling molecules Akt and RhoA are suppressed by NOV-002.

**Figure 4:**
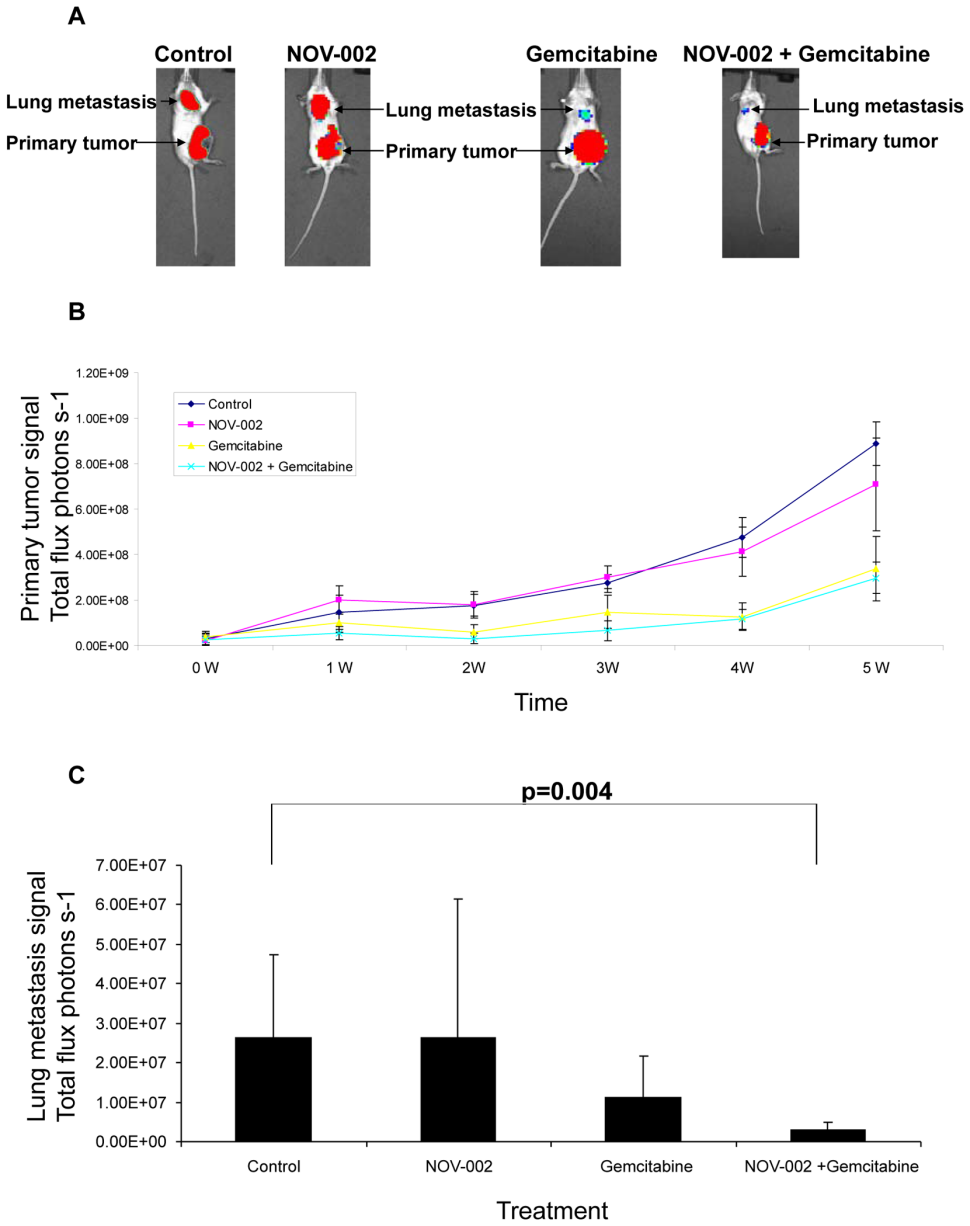
NOV-002 suppresses metastasis in combination with gemcitabine in a mouse model. (A) Bioluminescence imaging of lung metastasis and primary tumor. Images were taken five weeks following the treatment of control (PBS), NOV-002, gemcitabine or combination of NOV-002 and gemcitabine following the transplantation of breast cancer 4T1 cells in mammary fat pads. (B) Quantification of primary tumor growth with treatment of control (PBS), NOV-002, gemcitabine or combination of NOV-002 and gemcitabine following the transplantation of breast cancer 4T1 cells in mammary fat pads. (C) Quantification of metastasis growth after five weeks’s treatment of control (PBS), NOV-002, gemcitabine or combination of NOV-002 and gemcitabine following the transplantation of breast cancer 4T1 cells in mammary fat pads. 12.

The activity of NOV-002 on metastasis was determined in a mouse xenograft model. It has been shown that mammary tumor cell line 4T1 causes lung metastasis when it is transplanted in mammary fat pads, representing an effective orthotopic xenograft model for metastasis [27,38]. ErbB2, PI3K, Akt and RhoA are expressed in 4T1 cells (data not shown), which also makes this cell line suitable for our study. Gemcitabine, a nucleoside analog, was chosen for combinatorial therapy with NOV-002 because it is used in the treatment of multiple cancer types such as non-small cell lung cancer, breast cancer, pancreatic cancer and bladder cancer. 4T1 cells stably expressing a luciferase were used for the transplantation and luciferase signals detected by bioluminescence Xenogen system were used for the quantification of primary tumor and metastasis growth [27]. NOV-002 alone does not affect primary tumor or metastasis growth (Figures 4A, 4B and 4C) when compared with control treatment. Gemcitabine reduced primary tumor and metastasis growth by approximately 62% and 58% respectively five weeks after treatment when compared with control treatment (Figures 4B and 4C). NOV-002 in combination with gemcitabine suppressed primary tumor growth and metastasis by approximately 66% and 89% respectively when compared with control (Figures 4B and 4C). Although combinatorial treatment NOV-002 and gemcitabine showed no statistical difference on primary tumor growth when compared with gemcitabine treatment alone, the combination significantly suppressed metastatic tumor growth (Figures 4A and 4C). These results indicate that in combination with gemcitabine, NOV-002 suppresses metastasis in the mouse model.

## Discussion

The results of our study indicated that NOV-002 suppresses ErbB2 phosphorylation, PI3K activation and their downstream targets AKT and RhoA, which regulate cell invasion and metastasis (Supplementary Figure 7). Further studies are underway to investigate whether RhoA/ROCK/LIMK/Cofilin signaling pathway downstream of RhoA and β-catenin activation downstream of AKT play important roles in the control of cell invasion and metastasis. It is possible that NOV-002 suppresses invasion and metastasis by regulating signaling molecules and pathways in addition to ErbB2 and PI3K. Further studies are needed to identify these molecules and pathways.

NOV-002 is a mimetic glutathione disulfide that can regulate redox homeostasis. Studies suggest that oxidized glutathione does not passively enter cell membrane [29]. Thus it is likely that the effect of NOV-002 on cell functions is mediated by the impact of GSSG on cell surface proteins. ErbB2 is a surface receptor that could potentially be one of the targets GSSG affects. Studies of whether ErbB2 is modified by glutathiolation and its impact on ErbB2 functions are currently underway. It is highly possible that GSSG affects additional cell surface proteins. It is imperative to develop systemic methods to identify these targets and study the mechanisms that GSSG alters the functions of its target proteins.

Although NOV-002 suppresses ErbB2 and PI3K activation in vitro, NOV-002 alone, which has no cytotoxicity even at high dosage, has no effect on primary tumor or metastasis growth in the mouse xenograft model. It is possible that tumor cells use alternative signaling pathways to compensate for the partial inactivation of ErbB2-PI3K pathway by NOV-002. Alternatively, ErbB2-PI3K pathway is not a major signaling pathway responsible for the metastatic phenotype of 4T1 cells. It is possible that ErbB2-PI3K pathway becomes a dominant pathway for metastasis only after cells undergo stress such as gemcitabine treatment, which may explain the effectiveness of NOV-002 in metastasis suppression in combination with gemcitabine. Although clinical trial of NOV-002 in non-small cell lung cancer failed to show efficacy of survival improvement, it is possible that it can be effective in late stage cancer patients in combination with other chemotherapies.

Redox regulation is one of the critical aspects in cancer treatment. Cancer cells developed various mechanisms to cope with oxidized stress. It is possible that metastatic cells evolve to utilize different mechanisms than cells in the primary tumor to balance cellular redox. Therapies that change the balance of redox regulation in metastatic cancer cells may benefit metastasis treatment. Development of such therapies may aid the treatment of this deadly disease.

## Supplementary Material

Supplementary
