# Supplementary material for: NOV-002, A Glutathione Disulfide Mimetic, Suppresses Tumor Cell Invasion and Metastasis
Source: J Carcinog Mutagen. Author manuscript; Available in PMC 2013 Dec 26. (PMC3872994; doi:10.4172/2157-2518.S7-002)
Supplement: Supplementary [file NIHMS527565-supplement-Supplementary.zip › SuppelementoryFigure legends.docx]

**Supplementary Figure 1.** NOV-002 suppresses invasion in A549 and Colo205 cells. Invasion assay was used to quantify the number of invasive cells. Each panel represented stained invasive cells.

**Supplementary Figure 2.** NOV-002 treatment does not affect cell viability. Cell viability was quantified by MTT assay after NOV-002 or control treatment.

**Supplementary Figure 3.** Quantification of band intensity in immunoblots. The Western blots of ErbB2, pErbB2, P85 and pP85 were scanned and densities of bands were measured by Image-Pro Plus version 6.0 software. Data represent mean ± SD of three independent experiments.

**Supplementary Figure 4.** Knockdown of ErbB2 expression by shRNAs. Immunoblots of ErbB2 expression in Colo205 and A549 cells following the introduction of two independent ErbB2 shRNAs or control non-target shRNA.

**Supplementary Figure 5.** Knockdown of ErbB2 decreases invasion in Colo205 and A549 cells. Invasion assay was used to quantify the number of invasive cells. Each panel represented stained invasive cells.

**Supplementary Figure 6.** Quantification of band intensity in immunoblots. The Western blots of Akt, pAkt, RhoA and active RhoA were scanned and densities of bands were measured by Image-Pro Plus version 6.0 software. Data represent mean ± SD of three independent experiments.

**Supplementary Figure 7.** The scheme of NOV-002 functions in cell invasion and metastasis.
